# Supplementary material for: Effectiveness and Safety of Ayurvedic Medicines in Type 2 Diabetes Mellitus Management: A Systematic Review and Meta-Analysis
Source: Front Pharmacol. 2022 Jun 8;13:821810. doi: 10.3389/fphar.2022.821810 (PMC9213670; doi:10.3389/fphar.2022.821810)
Supplement: Supplementary file 1 [file DataSheet1.zip › Supplementary Material/Forest and Funnel Plots/Trigonella foenum-graecum/Sensitivity analyses/Trigonella foenum-graecum_FBG_sensitivity analysis.pdf]

Trigonella foenum-graecum - FBG - sensitivity analysis

Study	Intervention N	Intervention Mean	Intervention SD	Comparator N	Comparator Mean	Comparator SD	Mean Diff. with 95% CI	Weight (%)
Gupta 2001	12	119.90	25.00	13	113.00	36.00	6.90 [-17.60, 31.40]	5.90
Lu 2008	46	122.33	25.22	23	150.97	24.50	-28.64 [-41.15, -16.13]	8.88
Rafraf 2014	44	136.00	46.44	44	158.00	64.90	-22.00 [-45.58, 1.58]	6.11
Kaur 2016	30	141.23	10.11	30	144.43	9.86	-3.20 [-8.25, 1.85]	10.42
Singh 2016	20	121.55	12.63	20	113.70	12.99	7.85 [-0.09, 15.79]	9.93
Verma 2016	77	135.70	44.87	77	151.12	48.11	-15.42 [-30.11, -0.73]	8.32
Ranade 2017	30	151.08	28.88	30	158.57	27.46	-7.49 [-21.75, 6.77]	8.43
Gholaman 2018	10	185.40	17.25	10	216.10	26.32	-30.70 [-50.20, -11.20]	7.08
Kandhare 2018	59	162.80	50.72	53	170.05	57.45	-7.25 [-27.28, 12.78]	6.95
Hassani 2019	62	114.56	25.93	63	157.16	41.19	-42.60 [-54.69, -30.51]	8.98
Rashid 2019	32	113.50	7.93	32	134.58	5.40	-21.08 [-24.40, -17.76]	10.63
Hadi 2020	24	132.60	21.21	24	142.61	29.27	-10.01 [-24.47, 4.45]	8.38
**Overall**							-14.53 [-23.38, -5.68]	

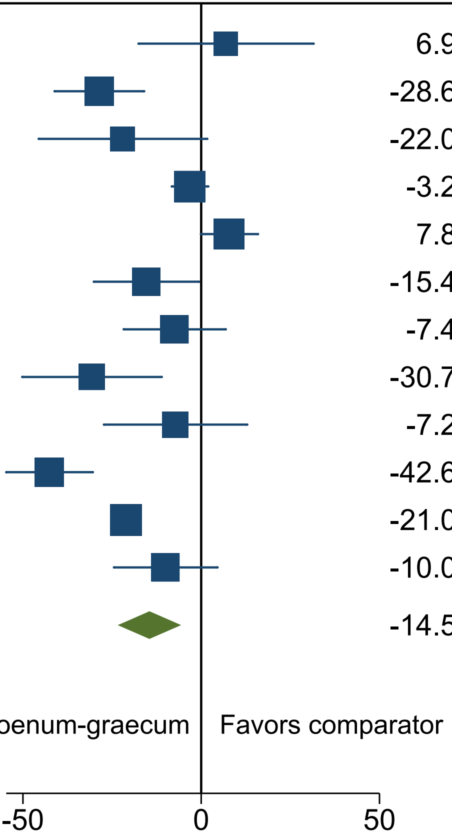

Heterogeneity: $\tau^2 = 188.77$, $I^2 = 89.24\%$, $H^2 = 9.29$

Test of $\theta_i = \theta_j$: $Q(11) = 98.13$, $p = 0.00$

Test of $\theta = 0$: $z = -3.22$, $p = 0.00$

Random-effects REML model
